# The autism-associated *Meis2* gene is necessary for cardiac baroreflex regulation in mice

**DOI:** 10.1038/s41598-022-24616-5

**Published:** 2022-11-23

**Authors:** J. Roussel, R. Larcher, P. Sicard, P. Bideaux, S. Richard, F. Marmigère, J. Thireau

**Affiliations:** 1 Present Address: Université de Montpellier, CNRS, Institut des Biomolécules Max Mousseron, Montpellier, France; 2 PhyMedExp, Université de Montpellier, INSERM, CNRS, CHRU de Montpellier, Montpellier, France; 3 IPAM, Platform for Non-Invasive Imaging in Experimental Models, Montpellier, France; 4 Institute for Neurosciences of Montpellier, Université de Montpellier, Inserm, Montpellier, France; 5 Institut de Génomique Fonctionnelle de Lyon (IGFL), École Normale Supérieure de Lyon, CNRS, Lyon, France

**Keywords:** Physiology, Cardiovascular biology

## Abstract

Recent understanding of Autism Spectrum Disorder (ASD) showed that peripheral primary mechanosensitive neurons involved in touch sensation and central neurons affected in ASD share transcriptional regulators. Mutant mice for ASD-associated transcription factors exhibit impaired primary tactile perception and restoring those genes specifically in primary sensory neurons rescues some of the anxiety-like behavior and social interaction defects. Interestingly, peripheral mechanosensitive sensory neurons also project to internal organs including the cardiovascular system, and an imbalance of the cardio-vascular sympathovagal regulation is evidenced in ASD and intellectual disability. ASD patients have decreased vagal tone, suggesting dysfunction of sensory neurons involved in cardio-vascular sensing. In light of our previous finding that the ASD-associated *Meis2* gene is necessary for normal touch neuron development and function, we investigated here if its inactivation in mouse peripheral sensory neurons also affects cardio-vascular sympathovagal regulation and baroreflex. Combining echocardiography, pharmacological challenge, blood pressure monitoring, and heart rate variability analysis, we found that *Meis2* mutant mice exhibited a blunted vagal response independently of any apparent cardiac malformation. These results suggest that defects in primary sensory neurons with mechanosensitive identity could participate in the imbalanced cardio-vascular sympathovagal tone found in ASD patients, reinforcing current hypotheses on the role of primary sensory neurons in the etiology of ASD.

## Introduction

Autism Spectrum Disorder (ASD) is the consequence of a neurodevelopmental defect affecting different nervous system structures and characterized by many diverse phenotypic manifestations including aberrant social interactions, repetitive behaviors, and restrictive interest. In addition, 90% of ASD patients are estimated to present sensory processing deficits, and an inability to elaborate appropriate behavioral responses due to impaired sound, touch, and sight perception^1^. This defective sensory perception can lead to an altered functional “vagal brake” associated with defective behavioral flexibility to stress^2^.

A large number of genes have been associated with ASD and are believed to be involved in various stages of building neuronal architecture, from neurogenesis to neurites outgrowth, synaptogenesis, and synaptic plasticity^3–6^. The diverse cellular expression and functions of ASD-associated genes across brain regions and neuronal cell types are reflected in the wide range of common and divergent phenotypic outcomes. Consequent to this genetic diversity, phenotypic characterization of the syndrome has often proven difficult, resulting in inconsistent conclusions. For instance, despite a paucity of information and conflicting findings in the literature, an imbalance between the sympathetic and parasympathetic branches of the autonomic nervous system is commonly observed in ASD patients^7–15^. Overall, these studies point to a lower autonomic nervous system activity suggested to likely result from a decreased parasympathetic activity. More strikingly, the characterization of autonomic activity in Rett syndrome, one of the most characterized ASD-related disorders, illustrates the diversity of the phenotypic manifestation of the vagal imbalance. Whereas some studies report a vagal imbalance with increased LF/HF ratio and HF component, others report a decreased cardiac baroreceptor sensitivity and cardiac vagal tone^7,16–20^. In the first case, it was suggested that individuals suffering from Rett syndrome have an increased sympathetic activity that is not counterbalanced by vagal tone, whereas in the second case, the authors concluded that Rett patients exhibit a low cardiovascular parasympathetic tone but a normal sympathetic activity. Nevertheless, in line with the current emerging hypothesis of the role of primary sensory neurons in the etiology of ASD, these observations raise the possibility that peripheral neurons in general and peripheral sensory neurons in particular are defective in some ASDs.

Recent advances in the understanding of ASD suggest that centrally affected neurons in ASD and peripheral touch mechanosensitive sensory neurons of the Dorsal Root Ganglia (DRG) share specific transcriptional programs regulating late neuronal differentiation^21,22^. These touch neurons express several of the ASD-linked genes and mutant mouse models for ASD exhibit primary sensory deficits. Specific inactivation of ASD-associated genes in the peripheral somatosensory system recapitulated some ASD symptoms such as altered cognitive and social behavior. Conversely, tissue-specific re-introduction of those genes in full knockout models not only rescued the normal functioning of primary touch neurons, but also some of the anxiety-like and altered social behaviors^21,22^. Thus, specific inactivation of ASD-associated genes allows the uncoupling of complex and intermingled ASD-associated symptoms.

Among the genes recently associated with ASD, the transcription factor (TF) *MEIS2* is a strong candidate to participate in the autonomic regulation of cardiac rhythm^23–25^. *MEIS2* is a member of the *MEIS* (Myeloid Ecotropic viral Insertion Site) family of homeobox TFs that belongs to the Three Amino-acid Loop Extension (TALE) family. These TFs are involved in the embryonic development of a plethora of organs and cell types, in particular in the nervous system^26,27^. In mice, Meis TFs are also strongly linked to heart embryonic development and postnatal functions^28–32^, and in humans, *MEIS2* haploinsufficiency causes severe neurodevelopmental defects with intellectual disability and ASD-like behavioral abnormalities, cleft palate and heart defects^23–25^. Combining mouse genetic and Heart Rate Variability (HRV) approaches, we previously showed that, independently of any heart morphological defects, specific *Meis1* inactivation in mouse developing sympathetic resulted in severe cardiac chronotropic incompetence eventually leading to sudden cardiac death^27^. This phenotype was attributed to a failure by sympathetic neurons to complete distal innervation of target organs, including the heart. Thus, combining conditional gene ablation in mice, HRV analysis and pharmacological testing of heart rate adaptation to blood pressure changes offers a powerful workflow to disentangle the mechanisms leading to cardiac dysautonia.

More recently, we found that specific *Meis2* inactivation in postmitotic peripheral sensory neurons dictates comparable phenotypes for DRG touch neurons with incomplete distal innervation, impaired electrophysiological responses to mechanical stimuli and reduced touch sensation^32^. In the present study, we hypothesize that *Meis2* targeted inactivation in peripheral sensory neurons interferes with the autonomic control of heart rhythms through the hemodynamic baroreflex, and independently of cardiac malformations. To this aim, we first used echocardiography to eliminate any possible heart morphology and contractility defects in our mutant mice. Secondly, using telemetric ECG recording in vigil non-anesthetized mice, we calculated ECG parameters (baselines and HRV analysis) from long-term signal recording. Using the same approach, we also characterized the cardiac responses for each genotype to several reference compounds (comparison before/after drug injection). Finally, to investigate how heart rhythm adapt to rapid hemodynamic change in the different genotypes, we simultaneously measured blood pressure and ECG in anesthetized mice, both in baselines condition and following injection of compounds known to modulate heart rate by activating the baroreflex activation. Our results showed that indeed *Meis2* expression in somatosensory neuron*s* is indispensable for the functional adaptation of cardiovascular parameters. These mutant mice exhibited increased sinus rhythm variability and modified sympathovagal index together with an altered cardio-inhibitory reflex (cardiac baroreflex) independently of any cardiac morphological and contractile defects. These results are consistent with the decreased cardiac baroreceptor sensitivity reported in ASD, and the decreased cardiac vagal tone and cardiac sensitivity to baroreflex in Rett patients, suggesting that suppressing *Meis2* function in late differentiating peripheral neurons recapitulates some of ASD symptoms.

## Results

### Meis2 mutant mice do not present any morphological or contractile heart defects

**Figure 1:**
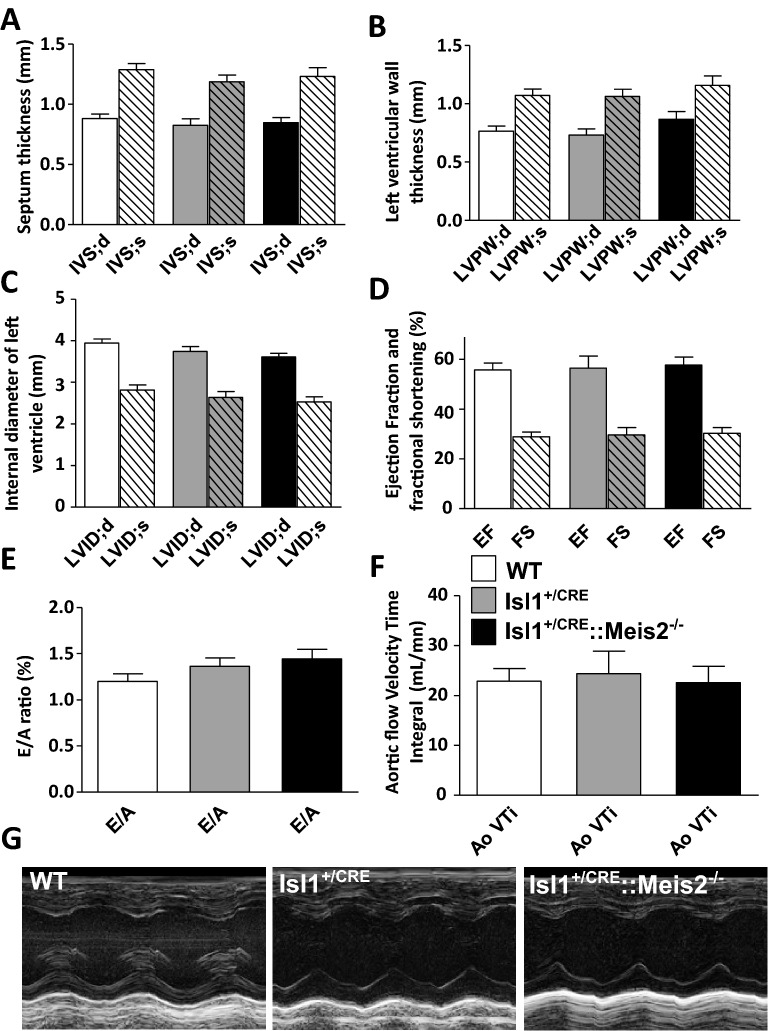
Isl1^+/CRE^::Meis2^LoxP/LoxP^ mice are devoid of morphological and contractile heart defects. Morphologic and left ventricular function parameters assessed by Doppler echocardiography in WT, Isl1^+/CRE^ and Isl1^+/CRE^::Meis2^LoxP/LoxP^ mice. Measures of IVS (**A**), LVPW (**B**), LVID (**C**), EF and FS (**D**) showed no difference between WT, Isl1^+/CRE^ and Isl1^+/CRE^::Meis2^LoxP/LoxP^ mice. Peak early (**E**) and late atrial contraction (**A**) mitral inflow wave velocities were measured and the E/A ratio was calculated (**E**) as the ascending aortic blood flow (**F**). (**G**) Representative M-modes images of Doppler echocardiography in WT, Isl1^+/CRE^ and Isl1^+/CRE^::Meis2^LoxP/LoxP^ mice. Abbreviations: IVS = thickness of the interventricular septum during diastole (d) and systole (s); LVPW = thickness of the posterior wall of the left ventricle during diastole (d) and systole (s); LVID = left ventricular internal diameter during diastole (d) and systole (s); EF (%) = Ejection Fraction in M-mode; FS (%) = Fractional Shortening in M-mode. Pulsed-wave Doppler of the ascending aortic blood flow was recorded permitting measurements of the velocity time integral (AoVTI). n = 9–11 mice in each group.

The mouse strain used here was Isl1^+/CRE^::Meis2^LoxP/LoxP^ in which the 8th homeobox-containing exon was flanked by LoxP sites^32^. Both in mice and humans, *Meis2* mutations cause severe developmental anomalies in the heart. Humans carrying heterozygous *MEIS2* missense mutations or 15q14 microdeletion involving *MEIS2* present a triad of cleft palate, atrial or ventricular septal heart defects, and developmental delay^23–25^. In another *Meis2*-null mouse strain, incomplete septation of the outflow tract known as persistent *truncus arteriosus* was reported, and specific Meis2 ablation in cardiac neural crest precursors led to a defective heart outflow tract^29^. Islet1 (Isl1) is also expressed in distinct cardiovascular lineages^33^ raising the possibility that CRE recombination in Isl1^+/CRE^::Meis2^LoxP/LoxP^ mice results in heart malformations. To ascertain whether the Isl1^+/CRE^::Meis2^LoxP/LoxP^ strain allows investigating cardiac autonomic function independently of heart malformations, we morphologically and functionally characterized the adult heart in WT, Isl1^+/CRE^ and Isl1^+/CRE^::Meis2^LoxP/LoxP^ mice using Doppler echocardiography (Fig. 1). Isl1^+/CRE^ mice were included as an additional control group to avoid misinterpretation due to Isl1 heterozygosity. Investigations of the parasternal long and short-axis views (Fig. 1) did not reveal any cardiac malformation. Thicknesses of the septum in systole (IVS;s; 1.29 ± 0.165, 1.19 ± 0.17, 1.23 ± 0.24 mm, respectively for WT, Isl1^+/CRE^ and Isl1^+/CRE^::Meis2^LoxP/LoxP^), in diastole (IVS;d; 0.88 ± 0.11, 0.83 ± 0.17, 0.84 ± 0.14 mm, respectively for WT, Isl1^+/CRE^ and Isl1^+/CRE^::Meis2^LoxP/LoxP^, Fig. 1A) were similar in all groups (*p* = 0.56). The left ventricular posterior wall in diastole (LVPW;d; 0.76 ± 0.14, 0.73 ± 0.15, 0.86 ± 0.21 mm, respectively for WT, Isl1^+/CRE^ and Isl1^+/CRE^::Meis2^LoxP/LoxP^, *P* = 0.35) and in systole periods (LVPW;s; 1.07 ± 0.17, 1.06 ± 019, 1.16 ± 027 mm, respectively for WT, Isl1^+/CRE^ and Isl1^+/CRE^::Meis2^LoxP/LoxP^, Fig. 1B) were identical in all groups (*P* = 0.45). Similarly, the left ventricle internal diameters were also indistinguishable among groups whatever the cardiac period (LVID;d and LVID;s; Fig. 1C). The ejection fraction (EF; 55.07 ± 9.3, 56.5 ± 14.4, 57.8 ± 10.5%, *P* = 0.89) and fractional shortening (FS; 28.9 ± 6.13, 29.6 ± 8.9, 30.3 ± 7.8%, *P* = 0.91), which are used as the conventional contractile function indexes, were also similar in WT, Isl1^+/CRE^, and Isl1^+/CRE^::Meis2^LoxP/LoxP^ mice (Fig. 1D). Finally, heart diastolic performances assessed by measuring left ventricle filling waves in standard 4 cavities view (E/A ratio; 1.20 ± 0.27, 1.32 ± 0.28, 1.44 ± 0.35, *P* = 0.367, Fig. 1E) and Aortic flow Velocity Time Integral (Ao VTI; 22.86 ± 8.37, 24.34 ± 13.54, 22.60 ± 10.65 mL/mn, for WT, Isl1^+/CRE^, and Isl1^+/CRE^::Meis2^LoxP/LoxP^ respectively, *P* = 0.92; Fig. 1F) did not show any difference attesting to normal hemodynamic parameters and contractile performances and suggesting that the outflow tract was not affected in Isl1^+/CRE^ and Isl1^+/CRE^::Meis2^LoxP/LoxP^ mice (Fig. 1G). To conclude, these observations exclude cardiac malformations or remodeling that may alter cardiac function, and allow thus investigating investigation of cardiac autonomic regulation independently.

### Meis2 mutant mice exhibit increased sinus rhythm variability and modified sympathovagal index

**Figure 2:**
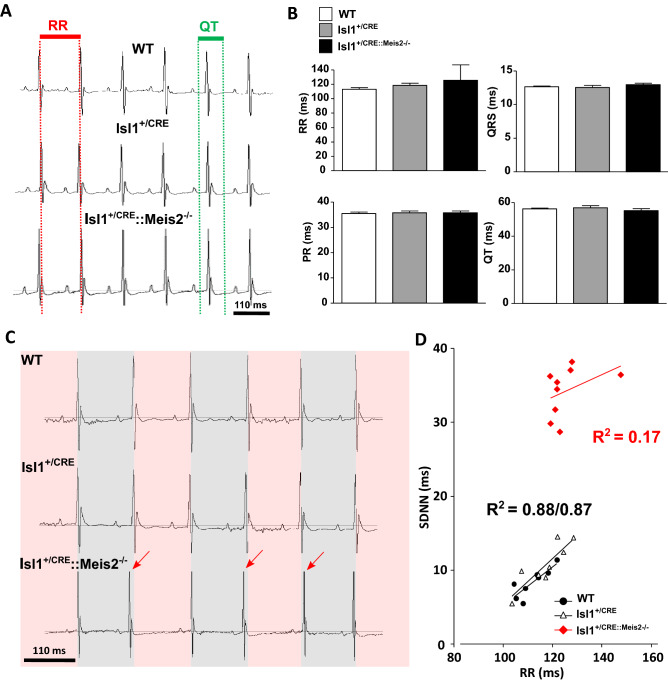
Increased sinus rhythm variability in Isl1^+/CRE^::Meis2^LoxP/LoxP^ mice. Telemetric recording of ECG in vigil WT, Isl1^+/CRE^ and Isl1^+/CRE^::Meis2^LoxP/LoxP^ mice in basal conditions. (**A**) Typical ECG traces for WT, Isl1^+/CRE^ and Isl1^+/CRE^::Meis2^LoxP/LoxP^ (**B**) Graphs showing the RR, PR, QRS and QT durations in the three groups of mice. No difference was observed between genotypes. n = 11–12 mice in each group. (**C**) Typical ECG traces of sinus variability monitored in WT, Isl1^+/CRE^ and Isl1^+/CRE^::Meis2^LoxP/LoxP^ mice. (**D**) Graph showing the correlation between SDNN and RR in the 3 groups of mice. n = 7–9 mice in each group. SDNN = Standard Deviation of normal to normal beat intervals.

**Figure 3:**
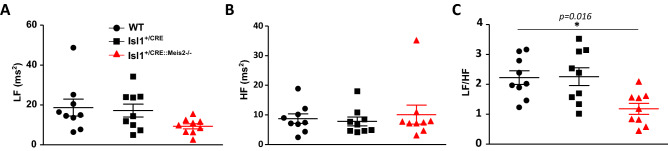
Decreased sympathovagal activity in Isl1^+/CRE^::Meis2^LoxP/LoxP^ mice. HRV analysis of WT, Isl1^+/CRE^ and Isl1^+/CRE^::Meis2^LoxP/LoxP^ vigil mice. Low-frequency band (LF) (**A**), High-frequency band (**B**) and sympathovagal index LF/HF (**C**) were obtained at baseline by fast Fourier transform and revealed a decrease of sympathovagal activity on heart rhythm. n = 7–9 mice in each group. *p ≤ 0.05 to WT.

We next characterized cardiac electrophysiological activity using telemetric electrocardiogram recording in *Meis2* mutant (Isl1^+/CRE^::Meis2^LoxP/LoxP^) and control (WT and Isl1^+/CRE^) mice. Using the telemetric system allows long-term recording of ECG on non-sedated and unrestrained mice. ECG analyses showed that the 3 strains exhibited comparable electrophysiological characteristics in baseline conditions (Fig. 2A,B; n = 11, 12, 11 in WT, Isl1^+/CRE^, and Isl1^+/CRE^::Meis2^LoxP/LoxP^, respectively). The mean values of the ventricular cycle length (RR in ms, 112.9 ± 5.9, 118.4 ± 8.7, 125.40 ± 8.87, for WT, Isl1^+/CRE^, and Isl1^+/CRE^::Meis2^LoxP/LoxP^ respectively, *P* = 0.42), the PR interval (interval between the onset of atrial depolarization until the beginning of the onset of ventricular depolarization in ms, 35.5 ± 1.5, 35.8 ± 1.80, 35.7 ± 2.1, for WT, Isl1^+/CRE^, and Isl1^+/CRE^::Meis2^LoxP/LoxP^ respectively, *P* = 0.36), the QRS (depolarization time of the right and left ventricles in ms, 12.6 ± 0.33, 12.5 ± 0.81, 12.9 ± 0.66 for WT, Isl1^+/CRE^, and Isl1^+/CRE^::Meis2^LoxP/LoxP^ respectively, *P* = 0.40) and of QT duration (time to depolarization-repolarization of ventricles in ms, 56.3 ± 1.4, 56.9 ± 3.4, 55.2 ± 3.6, *P* = 0.53) (Fig. 2B) were identical in all groups suggesting comparable cardiac conduction and depolarization/repolarization activities. No ectopic atrial or ventricular arrhythmia was detected in none of the 3 genotypes. By contrast, we observed a large sinus rhythm variability when *Meis2* was inactivated (Fig. 2C,D). Indeed, as showed by the SDNN assessing the total beat-to-beat variability of normal sinus beat, Isl1^+/CRE^::Meis2^LoxP/LoxP^ presented an increased variability (SDNN = 8.34 ± 0.68 ms for WT, SDNN = 10.68 ± 1.07 ms for Isl1^+/CRE^, and SDNN = 34.21 ± 1.12 ms for Isl1^+/CRE^::Meis2^LoxP/LoxP^, *p* = 0.001 and *p* = 0.002 vs WT and Isl1^+/CRE^ mice respectively), completed by a non-linearity to RR interval (R^2^ = 0.17) when compared to WT (R^2^ = 0.88) and Isl1^+/CRE^ (R^2^ = 0.87) mice (Fig. 2D). This could reflect a dysregulation of spontaneous beat-to-beat variability induced by autonomic pathways. We further assessed HRV by spectral analysis using Fast Fourier Transform (FFT) (Fig. 3). Low frequency (LF) was non-significantly decreased in Isl1^+/CRE^::Meis2^LoxP/LoxP^ mice using Anova test (LF = 18.7 ± 8.6, 17.2 ± 8.8, 9.31 ± 6.85 ms^2^, *p* = 0.3679, n = 9 in each group, Fig. 3A). High frequencies (HF) were similar in all groups (HF = 8.7 ± 2.8, 7.8 ± 2.4, 10.1 ± 4.6 ms^2^, *p* = 0.401, n = 9 in each group; Fig. 3B). However, the LF/HF ratio (2.2 ± 0.2, 2.3 ± 0.4, 1.18 ± 0.2, for WT, Isl1^+/CRE^, and Isl1^+/CRE^::Meis2^LoxP/LoxP^ respectively) was significantly decreased in Isl1^+/CRE^::Meis2^LoxP/LoxP^ mice compared to WT mice (*p* = 0.016, n = 9), and no difference was observed between WT and Isl1^+/CRE^ mice (Fig. 3C). These results obtained by spectral analysis suggest an alteration in the spontaneous control of cardiac rhythm by the autonomic system. Altogether, the large variability of sinus rhythm, the increase in SDNN and its non-linearity to RR, and the decrease of LF/HF ratio suggest an asymptomatic modification of spontaneous beat-to-beat adaptation in *Meis2* mutant mice and an overall decrease in sympathovagal activity.

### Meis2 mutant mice present non-typical heart rate adaptation with a blunt cardio-inhibitory reflex

**Figure 4:**
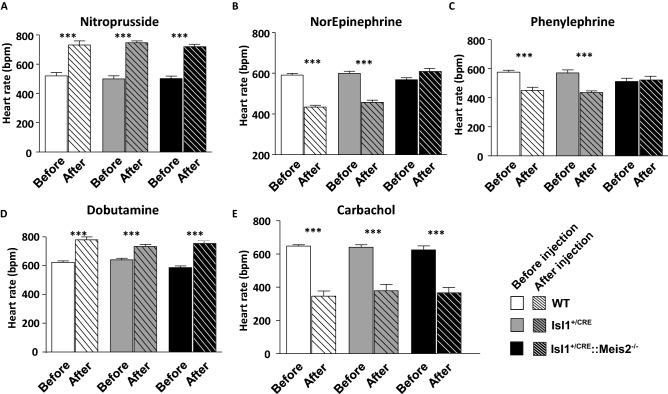
Blunted cardio-inhibitory reflex in Isl1^+/CRE^::Meis2^LoxP/LoxP^ mice. Graphs showing the heart rate adaptation after pharmacologically induced hemodynamic changes in WT, Isl1^+/CRE^ and Isl1^+/CRE^::Meis2^LoxP/LoxP^ vigil mice. Heart rate analyses were done before (plain bars) and after (dashed bars) injection of Nitroprusside (**A**), Norepinephrine (**B**), Phenylephrine (**C**), Dobutamine (**D**) and Carbachol (**E**). n = 5–7 mice in each group. ***p ≤ 0.001 vs before the injection.

To more clearly unmask the autonomic imbalance in Isl1^+/CRE^::Meis2^LoxP/LoxP^ mice, we challenged conscious transmitter-implanted mice with reference drugs. These drugs are well-known to induce fast hemodynamic changes that in turn activate cardiac rhythm adaptation reflexes^34–36^. After nitroprusside injection WT (from 519 ± 60 to 730 ± 70 bpm), Isl1^+/CRE^ (from 499 ± 51 to 745 ± 32 bpm) and Isl1^+/CRE^::Meis2^LoxP/LoxP^ (from 501 ± 46 to 719 ± 43 bpm) mice presented a reflex increase in heart rate (*p* < 0.001, n = 6, n = 6, n = 7 for WT, Isl1^+/CRE^ and Isl1^+/CRE^::Meis2^LoxP/LoxP^ animals respectively; Fig. 4A). By contrast, Isl1^+/CRE^::Meis2^LoxP/LoxP^ mice (567 ± 26 to 608 ± 36 bpm) challenged by norepinephrine injection failed to adapt compared to WT (from 590 ± 20 to 433 ± 24 bpm) and Isl1^+/CRE^ mice (from 599 ± 26 to 457 ± 25 bpm) that presented a large reflex-induced decrease of heart rate (n = 6, n = 6, n = 7 for WT, Isl1^+/CRE^ and Isl1^+/CRE^::Meis2^LoxP/LoxP^ animals respectively; Fig. 4B). Similarly, when mice were injected with phenylephrine (Fig. 4C), only Isl1^+/CRE^::Meis2^LoxP/LoxP^ mice (from 512 ± 54 to 523 ± 60 bpm, n = 6) failed to exhibit the expected heart rate decrease (from 575 ± 29 to 450 ± 49 bpm, n = 5 for WT and from 570 ± 48 to 435 ± 23 bpm for Isl1^+/CRE^, n = 5). These experiments demonstrate that WT and Isl1^+/CRE^ mice responded as expected to these pharmacological compounds, whereas in Isl1^+/CRE^::Meis2^LoxP/LoxP^ mutant mice, the cardio-inhibitory reflex was severely blunted.

Finally, to exclude a default in both cardiac adrenergic and muscarinic signaling pathways in Isl1^+/CRE^::Meis2^LoxP/LoxP^, we injected dobutamine and carbachol known to increase and decrease heart rate respectively by directly acting on cardiac tissues (Fig. 4D,E). As expected, in all groups of mice, dobutamine or carbachol injections similarly and significantly increased (from 622 ± 28 bpm to 779 ± 53 bpm, from 640 ± 28 to 733 ± 36 and from 587 ± 26 to 755 ± 45 bpm for WT, Isl1^+/CRE^, and Isl1^+/CRE^::Meis2^LoxP/LoxP^ respectively) or decreased (from 647 ± 20 to 345 ± 70 bpm, from 640 ± 34 to 378 ± 86 bpm, from 624 ± 54 to 364 ± 74 bpm, for WT, Isl1^+/CRE^, and Isl1^+/CRE^::Meis2^LoxP/LoxP^ respectively), the heart rate (*p* < 0.001). Altogether, these results demonstrate that despite functional adrenergic and muscarinic signaling pathways directly acting on cardiomyocytes, *Meis2* mutant mice were resistant to heart rate adaption when blood pressure was pharmacologically and acutely increased. By contrast, heart rate adapted normally to a pharmacologically induced rapid fall in blood pressure. These results suggest that *Meis2* inactivation interferes with cardio-inhibitory reflexes while cardio-excitatory reflexes remain unaffected.

### Meis2 is required for cardio-inhibitory reflex

**Figure 5:**
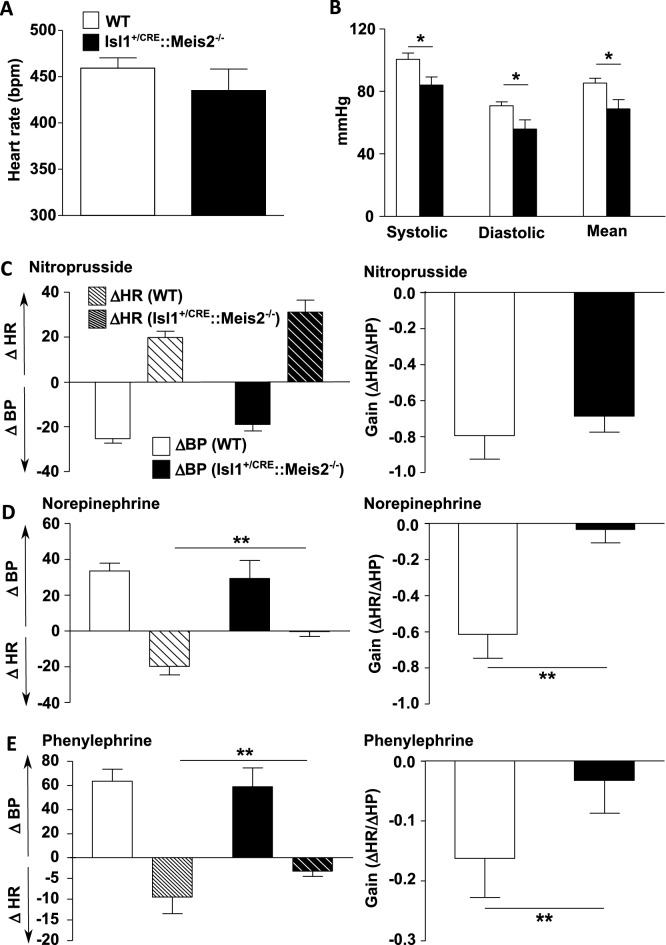
Lack of cardio-inhibitory reflex in Isl1^+/CRE^::Meis2^LoxP/LoxP^ mice. (**A**) Graphs showing the measurements of the heart rate (RR) in WT and Isl1^+/CRE^::Meis2^LoxP/LoxP^ anesthetized mice. (**B**) Graph showing measurements of blood pressure in WT and Isl1^+/CRE^::Meis2^LoxP/LoxP^ mice. The systolic, diastolic and mean blood arterial pressures at baseline are shown. (**C**–**D**) Graphs showing the variations in mean arterial blood pressure (BP), heart rate (HR) and related gain (ΔHR/ΔBP) following injection of Nitroprusside (**C**), Norepinephrine (**D**) and Phenylephrine (**E**). *p ≤ 0.05; **p ≤ 0.01 versus WT. n = 6–7 mice in each group. In (**C**), (**D**) and (**E**), plain bars indicate change of heart rate (ΔHR) and dashed bars represents modification in blood pressure (ΔBP). White bars indicate result in WT animal, black bars indicate results observed in Isl1^+/CRE^::Meis2^LoxP/LoxP^ mice.

Because *Meis2* mutant mice fail to activate cardio-inhibitory reflexes following injection of drugs that induce vasoconstriction, and to exclude a possible failure of norepinephrine or phenylephrine to evoke primary vasoconstriction, blood pressure was simultaneously monitored to heart rate in anesthetized mice before and after supramaximal dose injections of these compounds. We plotted the gain as the variation of heart rate over the variation of blood pressure (ΔHR/ΔBP; Fig. 5).

Because in the above telemetric experiments no differences were evidenced between control WT and Isl1^+/CRE^ mice, we focused our analysis by comparing WT and Isl1^+/CRE^::Meis2^LoxP/LoxP^ mice only. Under gaseous anesthesia, heart rate was not different between WT (462.4 ± 12.28 bpm) and Isl1^+/CRE^::Meis2^LoxP/LoxP^ animals (435.1 ± 16.63 bmp) before injection (*P* = 0.86). However, consistent with the low LF/HF ratio in Isl1^+/CRE^::Meis2^LoxP/LoxP^ animals (Fig. 2C), their basal systolic (83.92 ± 5.22 mmHg), diastolic (55.91 ± 5.85 mmHg) and mean blood arterial pressures (68.82 ± 7.76) were slightly but significantly lower than in WT mice (100.6 ± 3.904, 70.63 ± 2.65, 85.40 ± 3.027, respectively for systolic, diastolic and mean arterial pressure) (*p* ≤ 0.05, n = 15 in each group; Fig. 5A,B).

Following nitroprusside challenge, both WT and Isl1^+/CRE^::Meis2^LoxP/LoxP^ mice exhibited a similar decrease in mean arterial blood pressure leading to a rise in heart rate (Fig. 5C, Supplementary Fig. 1A and B) that ultimately resumed in a similar ΔHR/ΔBP gain (− 0.79 ± 0.26 vs − 0.68 ± 0.17, for WT and Isl1^+/CRE^::Meis2^LoxP/LoxP^ respectively, *P* = 0.88 n = 5 per group). When norepinephrine was injected, blood pressure largely increased in both WT (n = 6) and Isl1^+/CRE^::Meis2^LoxP/LoxP^ (n = 5) mice. However, whereas heart rate decreased in WT mice following norepinephrine injection, it remained stable in Isl1^+/CRE^::Meis2^LoxP/LoxP^ (Fig. 5D; Supplementary Fig. 1C,D), resulting in an almost null gain (ΔHR/ΔBP, − 0 to 03 ± 0.07) compared to WT (− 0.61 ± 0.14) (*P* = 0.0087). To confirm the absence of a cardio-inhibitory response provoked by the norepinephrine-induced vasopressor effect, another vasoconstrictor compound was tested. Thus, phenylephrine injection confirmed the lack of baroreflex activation in Isl1^+/CRE^::Meis2^LoxP/LoxP^ mice (n = 4 mice per group). While mean arterial blood pressure increased, the heart rate remained virtually unchanged in Isl1^+/CRE^::Meis2^LoxP/LoxP^ mice (Fig. 5E; Supplementary Fig. 1E,F). As a consequence the gain (ΔHR/ΔBP) in Isl1^+/CRE^::Meis2^LoxP/LoxP^ mice is quasi-null (− 0.03 ± 0.05) when compared to WT (− 0.162 ± 0.06 for WT) (*P* = 0.0098).

These results demonstrate that *Meis2* inactivation in *Isl1*-expressing cells impedes the cardio-inhibitory reflex that in normal conditions preserves cardiovascular homeostasis, whereas its counterpart, the tachycardic reflex, is normal. Both WT and Isl1^+/CRE^::Meis2^LoxP/LoxP^ mice presented functional adrenergic and muscarinic signaling pathways and developed the expected responses when blood pressure is challenged by vasodilator and vasoconstrictor drugs^37^, strongly supporting that heart and artery contractile activities are normal. Collectively our results suggest that rather than alteration of heart and/or arteries intrinsic functionality, the normal functioning of peripheral sensory neurons involved in this reflex is impaired.

### Neurons of the jugular-nodose complex are not lost following Meis2 inactivation

**Figure 6:**
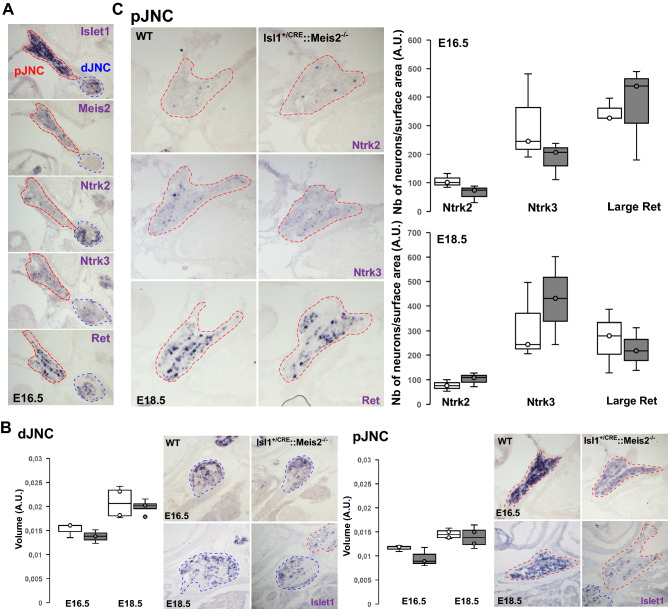
*Neurons of the Jugular-Nodose complex are not lost following Meis2 inactivation.* (**A**) Expression of Meis2 in the Jugular-Nodose complex of E16.5 mouse embryos. In situ hybridization (ISH) for *Meis2*, *Islet1* and other well-known markers for mechanosensitive neurons such as *Ntrk2*, *Ntrk3* and *Ret* on sagittal sections showed that *Meis2* is expressed by neuronal subpopulations in the proximal Jugular-Nodose complex (pJNC) in a pattern comparable with mechano-sensitive DRG neurons as previously described, but not in the distal Jugular-Nodose complex (dJNC). Red dotted lines delineate the pJNC and blue dotted lines delineate the dJNC. (**B**) Size Measurements of the pJNC and dJNC following ISH for Islet1 showed no change in the size of the ganglia at E16.5 and E18.5 of Isl1^CRE^::Meis2^LoxP/LoxP^ embryos compared to WT embryos (n = 3 for each genotype). Images are representative of the different ISH staining in the pJNC and dJNC. (**C**) Graphs showing the counting of Ntrk2, Ntrk3 and large Ret-positive neurons in the pJNC at E16.5 and E18.5. No changes were evidenced in the number of these neurons in Isl1^+/CRE^::Meis2^LoxP/LoxP^ embryos compared to WT (n = 3 for each genotype). Images are representative of the different ISH staining in the pJNC.

Our mouse model strikingly resembles a recently reported mouse model conditionally targeting Piezo channels in mechanosensitive neurons of the nodose ganglia and with abolished baroreflex^38^. In addition, *Meis2* and *Piezo2* are co-expressed in specific subpopulations of vagal neurons molecularly resembling mechanosensitive neurons of the DRG (Supplementary Fig. 2; https://ernforsgroup.shinyapps.io/vagalsensoryneurons/)^39^. A role of Meis2 in mediating target-field innervation raises the possibility that these neurons are lost during the naturally occurring neuronal death. To confirm Meis2 expression in vagal neurons and to investigate if its inactivation compromised their survival, we performed in situ hybridization for *Meis2* together with several classical identity markers for these mechanosensitive subclasses during (E16.5) and after (E18.5) naturally occurring neuronal death. A strong signal for *Islet1* mRNA was observed in the jugular-nodose complex (JNC) including the proximal (pJNC) and the distal (dJNC) parts of the complex (Fig. 6A). *Meis2* mRNA expression was mostly restricted to neuronal subpopulations located in the proximal complex together with subpopulations of neurons expressing Ntrk2, Ntrk3 or Ret. To investigate possible neuronal loss, we first measures dJNC and pJNC volumes on consecutive sagittal sections of E16.5 and E18.5 WT and Isl1^+/CRE^::Meis2^LoxP/LoxP^ embryos. Measures of dJNC at E16.5 in WT (0.0152 ± 0.0008 a.u.; n = 3) and Isl1^+/CRE^::Meis2^LoxP/LoxP^ (0.0138 ± 0.0008 a.u.; n = 3) were identical (Fig. 6B). Similarly, at E18.5, measures of dJNC at E18.5 in WT (0.0208 ± 0.0017 a.u.; n = 4) and Isl1^+/CRE^::Meis2^LoxP/LoxP^ (0.0199 ± 0.0008 a.u.; n = 4) were identical (Fig. 6B).

Measures of pJNC at E16.5 in WT (0.0116 ± 0.0004 a.u.; n = 3) and Isl1^+/CRE^::Meis2^LoxP/LoxP^ (0.0095 ± 0.0011 a.u.; n = 3) were identical (Fig. 6B). Similarly, at E18.5, measures of pJNC at E18.5 in WT (0.0146 ± 0.0005 a.u.; n = 4) and Isl1^+/CRE^::Meis2^LoxP/LoxP^ (0.0139 ± 0.0011 a.u.; n = 4) were identical (Fig. 6B). To confirm these results, we counted the numbers of Ntrk2-, Ntrk3 and large Ret-expressing neurons in pJNC and dJNC of WT and Isl1^+/CRE^::Meis2^LoxP/LoxP^ embryos at the same stages (Fig. 6C). The number of Ntrk2-, Ntrk3 and large c-Ret-positive neurons in E16.5 pJNC in WT (105.6 ± 14.2, 305.4 ± 89.2 and 349.4 ± 23.3 a.u. for Ntrk2, Ntrk3 and c-Ret respectively; n = 3) and Isl1^+/CRE^::Meis2^LoxP/LoxP^ (64.3 ± 17.4, 185.4 ± 38.5 and 368.8 ± 95.9 a.u. for Ntrk2, Ntrk3 and c-Ret respectively; n = 3) were identical. Finally, the number of Ntrk2-, Ntrk3 and large c-Ret-positive neurons in E18.5 dJNC in WT (76.4 ± 13.5, 315.5 ± 90.9 and 264.6.4 ± 75.2 a.u. for Ntrk2, Ntrk3 and c-Ret respectively; n = 3) and Isl1^+/CRE^::Meis2^LoxP/LoxP^ (102.3 ± 16.4, 425.8 ± 103.2 and 222.7 ± 50.4 a.u. for Ntrk2, Ntrk3 and c-Ret respectively; n = 3) were also identical. In conclusion, similar to what we observed for DRG neurons^32^, sensory neurons of the jugular-nodose complex are not lost following Meis2 inactivation.

## Discussion

In this work, we showed that specific inactivation of Meis2 TF in Isl1-expressing cells in mice severely impaired inhibitory baroreflex function independently of any developmental cardiac malformations or contractility defects of the heart and cardiovascular system. In addition, the *Meis2* expression in subclasses of vagal neurons that we and others reported and that are predicted to have proprioceptive and mechanosensitive properties^39^, together with the recent demonstration that Piezo2-positive vagal neurons are essential for the cardiac baroreflex^38^, strongly suggest that *Meis2* inactivation in those neurons could be responsible for the blunted inhibitory cardiac reflex we report. In this scenario, *Meis2*-expressing mechanosensitive neurons, including those from the JNG and possibly the DRG whose function is to sense stretch induced by artery and/or heart deformations fail to properly encode the information necessary to trigger the normal inhibitory baroreflex feedback. Thus, our model reinforces current hypotheses on alterations of primary sensory neurons function in ASD disorder^40^ and underlines the importance of conditionally targeted mouse models to disentangle intermingled and complex phenotypes found in human mutants.

The baroreflex is a classical and complex mechanism that coordinates adaptive cardio-vascular tone through both autonomic and sensory components^41^. Elevated blood pressure promptly triggers a compensatory decrease in cardiovascular output to maintain body and brain blood pressure within homeostatic ranges^42^. There is no real consensus about the sensory neuron subtypes involved. They are commonly called baroreceptors, display mechanosensitive properties, and project to precise locations on arteries where they sense arterial wall distortion. This arterial baroreceptor reflex system plays a dominant role in preventing short-term wide fluctuations of arterial blood pressure, as recurrently demonstrated in an experiment where arterial baroreceptor denervation leads to an increase of the beat-to-beat variability of blood pressure and related heart rate^43^.

The baroreflex is associated with some pathological conditions^41,44^, but only recently, the imbalance of cardiac autonomic regulation in patients with intellectual disabilities and ASD is emerging^7–15,45–48^. However, the origins of dysautonomia in ASD are still unclear and somehow controversial with highly variable profiles depending on the studies. Many studies report that ASD patients present a higher heart rate and that exposure to external stimuli leads to a blunted heart rate response compared to healthy subjects^48^. HR is increased in ASD patients compared to control due to a lower parasympathetic activity^46,47^, but other reports revealed on the contrary an increased parasympathetic activity^10^. Moreover, intermittent neuro-cardiovascular autonomic dysfunction affecting heart rate and blood pressure was also over-represented in ASD^14,49^.

Interpretation of results in human patients has proven complicated due to the genetic variability causing the different syndromes and the combinatory effect of multiple affected organs other than the nervous system. In most investigations related to cardiac autonomic regulation and HRV analysis in ASD, patients are rigorously matched in age and gender but cohorts usually do not take into account the genetic basis of the diagnosed ASD^8–15^. Indeed, the large number of neurodevelopmental genes supporting ASD symptoms, but also the variability of the symptoms accompanying different mutations within the same gene could account for discrepancies between studies. A good example linking gene dosage effect to the severity of phenotypic manifestation is Rett syndrome. Rett syndrome is associated with *MECP2* gene mutations, but the type of mutation, *i.e.* loss-of-function, gene duplication or triplication, and the degree of mosaicism for these mutations within cell types lead to highly heterogeneous phenotypic manifestations and clinical presentation ranging from microcephaly to normal brain size, shortened lifespan or not^50^. Nevertheless, studies have shown modified autonomic function both in children and adult ASD patients overall characterized by a lower autonomic nervous activity than healthy subjects.

The genetic links between ASD and congenital heart malformation in humans also prevent unmasking deleterious effects on cardiac autonomic regulation in ASD full knockout mouse models^51^. Recent advances in the understanding of the biology of the MEIS family of TFs and their well-known partners PBX members emphasized their essential contribution to cardiac morphogenesis and physiology. In humans, non-synonymous variants for *PBX1*, *PBX2*, *PBX3*, *MEIS1,* and *MEIS3* have been identified in patients with congenital cardiac defects^52^, and humans carrying *MEIS2* mutations present cardiac septal defects^23,24^. Similar phenotypes are also described in full-knockout models for those genes^29,30^. In mouse, genetic ablation of *Pbx1-3* at specific developmental stages lead to heart malformations^30^. *Pbx1* deficiencies result in persistent *truncus arteriosus*, whereas *Pbx2* and *3* inactivation leads to Pbx1 haploinsufficiency with the overriding aorta, ventricular septal defect, and bicuspid aortic valves^30^. *Meis1* and *Meis2* mutant mice also exhibit cardio-vascular and septal defects^27,29,30,53,54^.

Surprisingly, our Doppler-echocardiography investigations did not reveal any heart morphological or contractile defects. This might be due to a later *Meis2* inactivation in cardiac neural crest compared to the AP2α-IRES-Cre strain used by others^29^, at a time when *Meis2* is no longer required. Given that both genes are involved in heart morphogenesis, this might also result from redundant *Meis1* and *Meis2* activities within the timeframe of our genetic ablation. Nonetheless, we previously showed that *Meis1* ablation-induced septal defect depends on the CRE strain used for neural crest gene ablation. *Meis1* inactivation in early neural crest resulted in septal defects, but *Meis1* inactivation in late neural crest did not produce contractile and morphological defect^27^.

We also showed that at baseline conditions using long-period telemetric ECG recording, *Meis2* mutant mice do not present symptomatic heart rhythm disturbance such as altered heart rate or major sinus pause or arrest, or atrial/ventricular ectopic beats. Instead, large variability in sinus rhythm confirmed by high HRV, without brady- or tachycardia, was observed. A profound sinus node dysfunction in *Meis2* mutant is thus unlikely. We further demonstrated that the large beat-to-beat variability in *Meis2* mutant mice results from a dysregulation of the sensory-autonomic control of cardiac rhythm. We identified a lower sympathovagal activity at baseline reflected by the decreased LF/HF ratio that could also explain the low mean arterial pressure observed in mutant mice. When using drugs that rapidly and robustly modify blood arterial pressure, we unmasked a sensory-autonomic dysregulation characterized by a blunted cardio-inhibitory reflex. Surprisingly, only the cardio-inhibitory baroreflex was affected, but the sympathetic activation following a fall in blood pressure was maintained although both vasoconstriction and vasodilation could be pharmacologically elicited. We have to note that in these experiments designed to assess rapid changes in heart rate following pharmacological injections (Fig. 4), heart rates before dosing were quite different between conditions for the same genotype and when compared to baseline values obtained from long-term ECG recording. Such a difference in heart rate calculated from a limited ECG duration could result from the minor change in environmental conditions of housing/stress in the few hours preceding the ECG recording and drug administration in vigil mice.

According to the vagal dominance in the beat-to-beat baroreflex adjustment of HR and blood pressure, and the increased variability during baroreceptor denervation reported in several animal models^43^, we suggest a defective baroreceptor-related-vagal pathway induced by *Meis2* inactivation. Moreover, because *Meis2* is not expressed in sympathetic neurons^27^, along with the observation that basal mean heart rate is unaffected and the cardio stimulatory reflex seems to be unaltered, we can exclude that sympathetic nerves were affected by *Meis2* deletion.

Instead, we conclude that *Meis2* inactivation interferes with the sensory component of the vagal-mediated baroreflex. First, because of the possible *Meis2* recombination sites following CRE activity when using the Isl1^CRE^ strain. The LIM-homeodomain TF Isl1 is expressed by several neural and non-neural tissues both during embryonic development and postnatal life amongst which the peripheral and central nervous systems, the pancreas, the heart, and the pituitary gland^55–59^. However, the interaction of tissues other than the nervous system with the baroreflex is unlikely. Besides sensory and autonomic peripheral neurons, specific CNS neuronal populations express Isl1 including spinal motor neurons, retinal ganglion cells, hypothalamic, central amygdala, and striatal neurons^26,60–66^. We therefore cannot fully exclude that *Meis2* recombination also occurs in some of these central neuronal populations that somehow participate in the autonomic imbalance we report in Isl1^+/CRE^::Meis2^LoxP/LoxP^ mice.

Secondly, the large literature linking sensory neurons to baroreflex and our recent finding that *Meis2* is necessary for the normal functioning of peripheral mechanosensitive neurons place Meis2-expressing peripheral sensory neurons in the best position to support the lack of cardio-inhibitory reflexes in these mice. Besides the autonomic system that includes parasympathetic and sympathetic efferents and controls the heart and blood vessels contractility, peripheral sensory innervation of the heart is of dual origin^67,68^. Anatomically, sensory fibers originate from vagal neurons located in the jugular-nodose complex and run through the vagus and the inferior cardia nerve^68^. Afferent sensory fibers sense local target organ activities such as tissue tension and send the information to higher brain structures to elaborate an adapted response. Although most afferent and efferent information to the heart navigates through the vagus nerve^39,42^, there is evidence that DRG sensory neurons are also involved, in particular for cardiovascular reflexes^68–70^. Retrotracing experiments in cats, dogs, and rats injected at different locations in the heart, coronary artery, or the inferior cardiac nerve labeled neurons in the DRG indicate that the heart and arteries also receive afferent sensory fibers from the DRG^71–76^. In addition, molecular characterization of these neurons revealed that they express a range of markers compatible with the identity of several subclasses of DRG sensory neurons^75,77,78^.

Vagal neurons have been studied for a very long time, but the knowledge and understanding of the precise identities and physiological functions of the different subpopulations of vagal sensory neurons remain fragmented mainly because of the lack of molecular knowledge and tools to specifically target them. Nerve sectioning experiments combined with the mixed nature of the vagus nerve also impede the full interpretation of their precise function. Cranial ganglia contributing to the vagal nerves are multiple and arise from the neural crest-derived jugular ganglia and the placode-derived nodose and petrose complex that eventually merge during embryogenesis^79^. The molecular characteristics of these primary sensory neurons have only been very recently elucidated and showed that nodose and jugular neurons are molecularly fundamentally different with jugular neurons sharing many features with somatosensory DRG neurons^39^. In this scRNAseq study^39^, Meis2 was detected in 2 of the 18 nodose neuron clusters, and in 4 of the 6 jugular neurons clusters with relatively high expression in clusters displaying a molecular profile similar to myelinated DRG neurons involved in gentle touch. Functional classification of nodose clusters predicted *Meis2* expressing populations to have DRG proprioceptive-like features. Interestingly, in this database (https://ernforsgroup.shinyapps.io/vagalsensoryneurons/), the mechano-sensitive Piezo2 channel recently shown to be involved in baroreflex^38^ was coexpressed in all Meis2-expressing clusters (Supplementary Fig. 2). Thus, mechanosensitive neurons of the DRG and the Jugular-Nodose complex are molecularly highly similar.

In our mouse model, we could not evidence any neuronal loss of vagal neurons of the JNG in E16.5 and E18.5 embryos, suggesting that *Meis2* inactivation does not affect neuronal survival or identity as demonstrated by the normal expression of Ntrk2, Ntrk3 and Ret. Although we cannot fully conclude that these neurons are lost in adult mice, these results are in line with our previous studies on *Meis1* or *Meis2* inactivation in different types of peripheral neurons^27,32^. When *Meis1* is specifically inactivated in sympathetic neurons, distal innervation of target organs, including the heart, is compromised, early sympathetic specification is unaffected, and sympathetic neurons massively died concomitant to naturally occurring neuronal death^27^. More strikingly, using the very same mouse strain as in the present study, we found that mechanosensitive neurons of the DRG that normally express *Meis2* survived at adult stages but failed to fully differentiate and elaborate complex distal peripheral sensory terminals mediating touch sensation in the skin^32^. In both mouse models, these peripheral innervation defects result in physiological consequences: mice lacking *Meis1* in sympathetic neurons display severe chronotropic incompetence due to sympathetic dysfunction, and mice lacking *Meis2* in DRG mechanosensitive neurons have impaired touch sensations. Using another conditional *Meis2* strain, Machon et al. inactivated *Meis2* in the neural crest, including the neural crest-derived cranial sensory ganglia encompassing trigeminal (V), facial (VII) and vestibulocochlear (acoustic) nerves (VIII)^29^. Although the authors did not thoroughly detail their findings, most neural crest-derived cranial ganglia were reported to be present, but nerves exiting the ganglia seemed less numerous and less ramified than in WT embryos as seen by whole mount neurofilament staining. However, in this study, the physiological consequences have not been investigated.

To conclude, although we could not unambiguously demonstrate that Meis2 expressing vagal and/or DRG neurons are directly responsible for the blunted autonomic response and the lack of baroreflex in Isl1^+/CRE^::Meis2^LoxP/LoxP^ mice, our study clearly showed that our genetically modified animal model is a very appropriate tool to study autonomic dysregulation independently of cardiac remodeling. Not only our results provide an additional example of an ASD-associated gene which mutation in mouse impairs primary sensory function, but extend its consequences on the regulation of the cardio-vascular system. It is thus possible that primary sensory defects are more commonly associated to ASD than previously believed. In the future, it will be of interest to investigate if other ASD mouse models with known deffective mechanosensitive “touch” neurons” such as Mecp2, Shank3b or Fmr1^22,40^ also exhibit similar sensory defects that influence cardio-vascular homeostasis. For instance, a study in Mecp2 mutant mice reports a normal baroreflex^80^, suggesting that impaired primary touch function not necessarily associate to blunted cardiac baroreflex. Nevertheless, these observations in mouse emphasize the importance of considering the genetic basis of ASD in studies on sensory and autonomic functions in patients. Finally, in addition to the skin, primary mechano-sensitive neurons also project to most internal organs and participate to their homeostasis^81–83^, raising the possibility that some ASD mouse models and ASD patients also exhibit primary sensory defects with consequence on other internal organs that the cardio-vascular system.

## Material and method

### Ethical statement

All protocols complied with Directive 2010/63/EU of the European Parliament and the Council of 22 September 2010 for the protection of animals used for scientific purposes. All experimental protocols were approved by the Ethics committee for animal experiments, Languedoc Roussillon, C2EA-36 (agreement: B34-172-38; project APAFIS#11026). All methods are reported in accordance with ARRIVE guidelines (https://arriveguidelines.org).

### Animals

All efforts were made to minimize animal suffering during the experiment and to reduce the number of animals used by performing echocardiography and ECG recording in the same animal when possible, and for pharmacological dosing (2 days of washing period). Animals were housed in a temperature-regulated room (12 h day/12 h night cycle) with ad libitum access to food and water. Protocols were only conducted by trained and authorized experimenters.

The two genetically modified strains of mice used in this study have previously been reported^32,33^. Mice of three different genotypes were analyzed in the present study for in vivo experiments, wild-type (WT, n = 15, weight = 22.9 ± 1.8 g), Isl1^+/CRE^ (n = 10, weight = 20.2 ± 1.9 g) and Isl1^+/CRE^::Meis2^LoxP/LoxP^ strain (n = 15, 21.4 ± 1.7), aged 3 months, randomly assigned (equal proportion of males and females).

### Echocardiography

To decipher whether Meis2 is involved in morpho-functional changes in cardiac structures, we fully characterized the morphology and the contractile function of the heart using the ultrasonic method. Mice were anesthetized with 1.5% isoflurane in 100% oxygen to reach a comparable heart rate and placed on a heating table in a supine position. Having a comparable heart rate allows us to compare parameters that depend on the heart rate. Body temperature was monitored through a rectal thermometer to be maintained at 36–38 °C and ECG was recorded all along the echocardiographic procedure with limb electrodes. Contractile functions were assessed through the ejection fraction (EF%) and the fractional shortening (FS%). EF and FS were calculated from the left ventricular internal diameters (LVID) on M-mode measurements at the level of papillary muscles in a parasternal short-axis two-dimensional view using Vevo 2100 (VisualSonics, FujiFilm, Netherlands). To better consider left ventricular morphology and possible outflow tract remodeling or malformation, EF was also calculated from a B-mode parasternal long-axis view (EF% B-mode) by tracing endocardial end-diastolic and end-systolic borders to estimate left ventricular volumes, and the endocardial fractional area change (FS%) on a parasternal short-axis view at papillary muscle level was similarly measured. Mitral flow was recorded by a pulsed-wave Doppler sampling at the tips of the mitral valve level from the apical four-chamber view. Peak early (E) and late atrial contraction (A) mitral inflow wave velocities were measured and the ratio E/A was calculated. Pulsed-wave Doppler of the ascending aortic blood flow was recorded permitting measurements of the velocity time integral (AoVTI). To assess the morphology of the heart, the septum thickness, the left ventricular wall thickness, and the internal diameter of the left ventricle were measured. All measurements were quantified and averaged for three cardiac cycles as previously done^27^.

### ECG in conscious mice

To assess the electrical function of the heart of each genotype and characterize their heart rhythm, ECG signals were recorded and then analyzed with dedicated software, first from long period ECG (12 h) and then using short sequence (2 h) of signal for pharmacological testing.

#### Long-term recording

Electrocardiogram (ECG) was monitored by a telemetry system on vigil unrestrained mice. After a pre-anesthesia (physical) evaluation, the transmitter (PhysioTel, ETA-F10 transmitter) was inserted in mice subcutaneously along the back under general anesthesia (2% inhaled isoflurane/O2, Aerrane, Baxter, France) coupled with local anesthetic (lidocaine 0.5%), and two ECG electrodes were placed hypodermically in the region of the right shoulder (negative pole) and toward the lower left chest (positive pole) to approximate lead II of the Einthoven surface ECG. During the procedure, respiratory and cardiac rate and rhythm, adequacy of anesthetic depth, muscle relaxation, body temperature, and analgesia were monitored to avoid anesthesia-related complications. Post-operating pain was considered during the one-week post-implantation period and buprenorphine (0.3 mg kg^−1^ sc) could be done at case per case. ECG monitoring was performed 2 weeks after recovery from surgery in the home cage with a signal transmitter–receiver (RPC-1) connected to a data acquisition system (Ponemah system, Data Sciences International, Saint Paul, USA). Data were collected continuously over 24 h at a sampling rate of 2000 Hz as previously^84^.

#### Short-term (pharmacological) recording

ECG recordings were also performed on un-anesthetized mice to evaluate drug effects. For this, we proceeded to pharmacological injection of Nitroprusside (2.0 mg k^−1^ ip, NaCl, 0.9%), Norepinephrine (2.5 mg kg^−1^ ip, NaCl 0.9%), Phenylephrine (2.5 mg kg^−1^ ip, NaCl 0.9%), Dobutamine (1 mg kg^−1^ ip) and Carbachol (0.5 mg kg^−1^ ip, NaCl 0.9%) according to literature^34,85,86^. All molecules were purchased at Sigma-Aldrich (France) and diluted in NaCl sterile solution (Aguettant, France). Sodium Nitroprusside is a major vasodilator by acting on NO release and induces a pronounced reflex tachycardia. Norepinephrine is the neurotransmitter released by postganglionic neurons of the sympathetic system (α1 and β1 adrenergic receptor agonist) inducing major hypertension followed by reflex-bradycardia. Phenylephrine is a specific α1-adrenergic receptor agonist, increasing peripheral resistance and blood pressure that precipitates in sinus bradycardia due to vagal reflex. Dobutamine is a sympathomimetic, mainly through β1 adrenoreceptors activation leading to a rapid rise in heart rate by acting directly on cardiomyocytes. Carbachol is a nonselective muscarinic receptor agonist leading to profound direct bradycardia. Pharmacological experiments were conducted according to a cross-over design with a washout period of 48–72 h at minimum between dosing sessions.

#### ECG waveforms analysis of long-period ECG signal

Continuous digital recordings were analyzed offline after being digitally filtered between 0.1 and 1000 Hz. ECGs during nocturnal periods (12 h) and pharmacological testing were analyzed with Ponemah software using template automatic detection of PQRST, secondly validated by an operator. In addition, the presence of potential ectopic beats was scanned by hand. The mean RR interval and the mean PR, QRS, and QT durations were exclusively calculated from the sinusal beats, excluding artifacts and arrhythmia during the nocturnal period. The QT interval was defined as the time between the first deviation from an isoelectric PR interval until the return of the ventricular repolarization to the isoelectric TP baseline from lead II ECGs^34^.

#### Analysis of autonomic influence on heart rate from long period ECG signal

To assess the sympathetic and vagal influences on heart rhythm, we performed an HRV analysis. This method is based on the variations of a cardiac period of successive ‘beat-to-to-beat’ heart rate also named spontaneous heart rate. Indeed, the autonomic nervous system adapts continuously heart rate to metabolic needs, inducing beat-to-beat heart rate variability by modifying the automatic sinus activity through a complex interplay of the ortho-sympathetic and parasympathetic (or vagal) systems. Time- and frequency domain indices of HRV are the standard parameters to evaluate ANS activity as well in clinics as in fundamental research. Total variability was assessed with the standard deviation of all normal RR intervals (SDNN) in the time domain. HRV was also evaluated by power spectra analysis (ms^−2^) using the fast Fourier transformation (segment length of 2048 beats, linear interpolation with resampling to a 20-Hz interbeat-time series, and Hamming windowing). The cut-off frequency ranges for the low-frequency (LF: 0.15–1.5 Hz) and high-frequency powers (HF: 1.5–5 Hz) were chosen according to those used in the literature. As in humans, the low frequency (LF) reflects a complex interaction between sympathetic and parasympathetic ways that modulate heart rate including baroreflex function^87^. The efferent vagal activity rests as the major contributor to the HF component, as seen in clinical and experimental observations of autonomic maneuvers such as electrical vagal stimulation, muscarinic receptor blockade, and vagotomy. Thus, as previously performed^27,88^, the cardiac sympathetic and spontaneous baroreflex activities were assessed from LF, the vagal activity was assessed from HF and the LF/HF ratio, conjointly with the mean values of HF and LF power, was used to assess sympathovagal activity on heart rhythm.

#### ECG waveforms analysis during pharmacological testing

Specific to pharmacological testing, parameters were measured 2 h before and after injection. Effects were estimated by comparing values obtained before administration to values obtained at stable-maximum responses i.e. between 2 and 10 min post-dosing, depending on the drugs as previously reported^34,88^. A minimum of 15 complexes on a stable period were used for analysis and averaged. All experiments with drugs were performed between 7:00 am and 9:00 am. within less than 30 min in each study.

### BP and HR recordings under anesthetized conditions

To determine the origin of response failure of some molecules observed by telemetry, we recorded blood pressure coupled to heart rate change in anesthetized animal (2% inhaled isoflurane/O2, Aerrane, Baxter, France) using Powerlab system and LabChart software (Blood pressure module; ADInstruments Ltd, France). To this aim, a Millar Mikro-Tip^®^ pressure catheter is introduced in the carotid to record arterial blood pressure, and diastolic, systolic, and mean arterial blood pressures were calculated. In parallel, ECG was recorded using lead II Einthoven derivation using micro-needles to assess the heart rate changes induced by hemodynamic modifications. Parameters were measured in baseline conditions and after injection of Nitroprusside (2.0 mg k^−1^ g ip, NaCl, 0.9%), Norepinephrine (2.5 mg kg^−1^ ip, NaCl 0.9%) and Phenylephrine (2.5 mg kg^−1^ ip, NaCl 0.9%). Parameters were measured and averaged during the maximal response, on fifteen complexes. The delta of heart rate and delta of mean BP was calculated. The Gain (Delta HR/Delta BP) was done and reflects cardiovascular adaptation during pharmacological dosing through baroreflex. All experiments were conducted between 7:00 am and 11:00 am.

### In situ hybridization

RNA probes used in the study and in situ hybridization procedures have been reported previously^27,32^. Briefly, tissues were collected and fixed in 4% paraformaldehyde/PBS overnight at 4 °C and incubated overnight at 4 °C for cryopreservation in increasing sucrose/PBS solutions (10–30% sucrose). After snap freezing in TissueTek, embryos were sectioned at 14-µm thickness and stored at − 20 °C until use. Before hybridization, slides were air-dried for 2–3 h at room temperature. Plasmids containing probes were used to synthesize digoxigenin-labeled or fluorescein-labeled antisense riboprobes according to the supplier’s instructions (Roche) and purified by LiCl precipitation. Sections were hybridized overnight at 70 °C with a solution containing 0.19 M NaCl, 10 mM Tris (pH 7.2), 5 mM NaH2PO4*2H2O/Na2HPO4 (pH 6.8), 50 mM EDTA, 50% formamide, 10% dextran sulphate, 1 mg/ml yeast tRNA, 1XDenhardt solution and 100–200 ng/ml of probe. Sections were then washed four times for 20 min at 65 °C in 0.4X SSC pH 7.5, 50% formamide, 0.1% Tween 20 and three times for 20 min at room temperature in 0.1 M maleic acid, 0.15 M NaCl and 0.1% Tween 20 (pH 7.5). Sections were blocked for 1 h at room temperature in presence of 20% goat serum and 2% blocking agent (Roche) before incubation overnight with AP-conjugated anti-DIG-Fab-fragments (Roche, 1:2000). After extensive washing, hybridized riboprobes were revealed by performing an NBT/BCIP reaction in 0.1 M Tris HCl pH 9.5, 100 mM NaCl, 50 mM MgCl2 and 0.1% Tween 20. Wide-field microscopy (Leica DMRB, Germany) was used to take the images.

### Statistical analysis

All values are expressed as means ± SEM. For data from more than two experimental groups, one-way or two-way ANOVA was used to assess group means followed by the Bonferroni posthoc test. Paired comparisons were made if needed. *P* ≤ 0.05 was taken to denote statistical significance.

## Supplementary Information

Supplementary Information 1.

Supplementary Information 2.

Supplementary Legends.
