# Supplementary figures and images for: The autism-associated Meis2 gene is necessary for cardiac baroreflex regulation in mice
Source: Sci Rep. 2022 Nov 23;12:20150. doi: 10.1038/s41598-022-24616-5 (PMC9684552; doi:10.1038/s41598-022-24616-5)

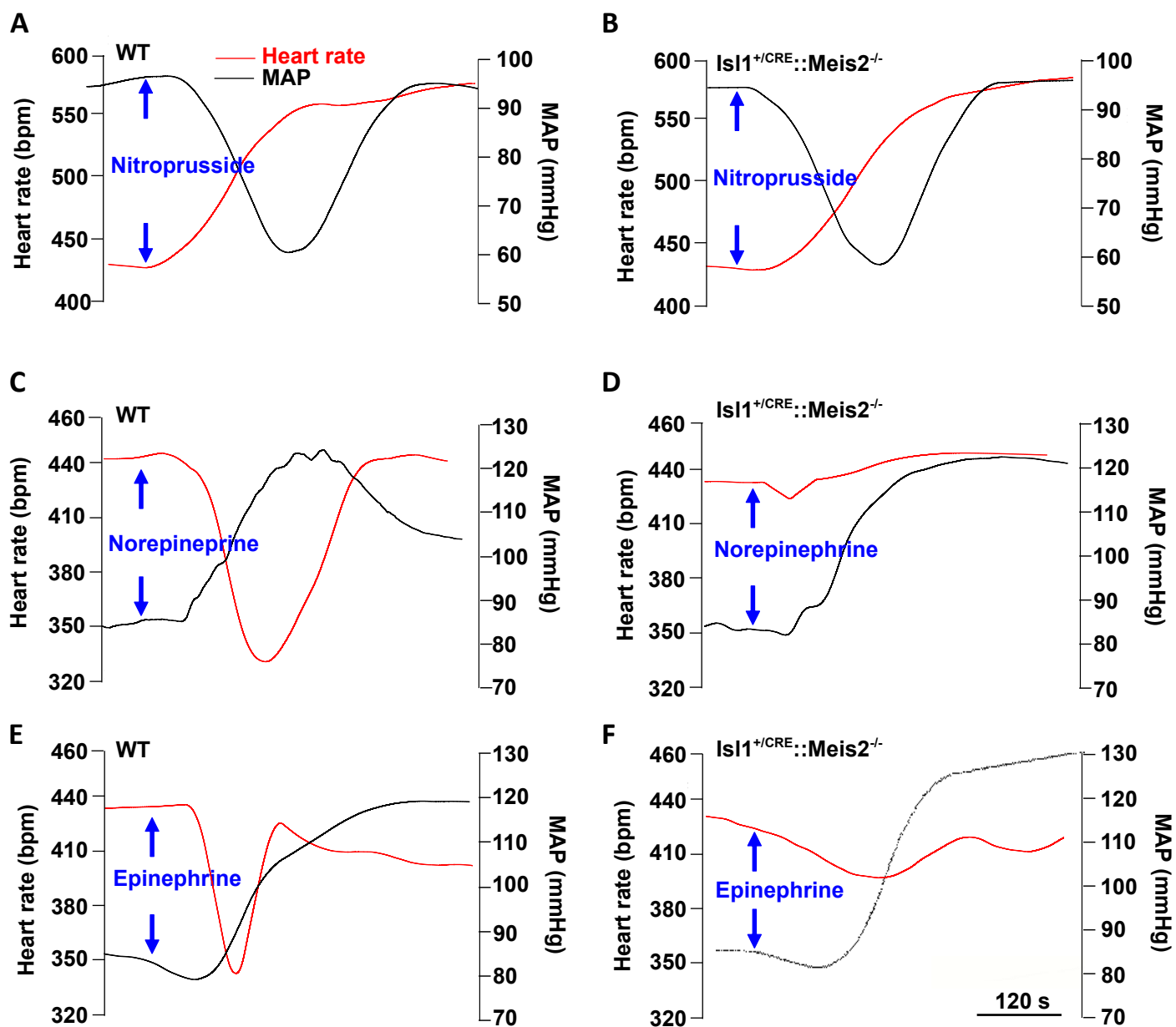

**Figure Supplementary 1**

Supplement: Supplementary file 1 — Supplementary Information 1. [file 41598_2022_24616_MOESM1_ESM.pdf]

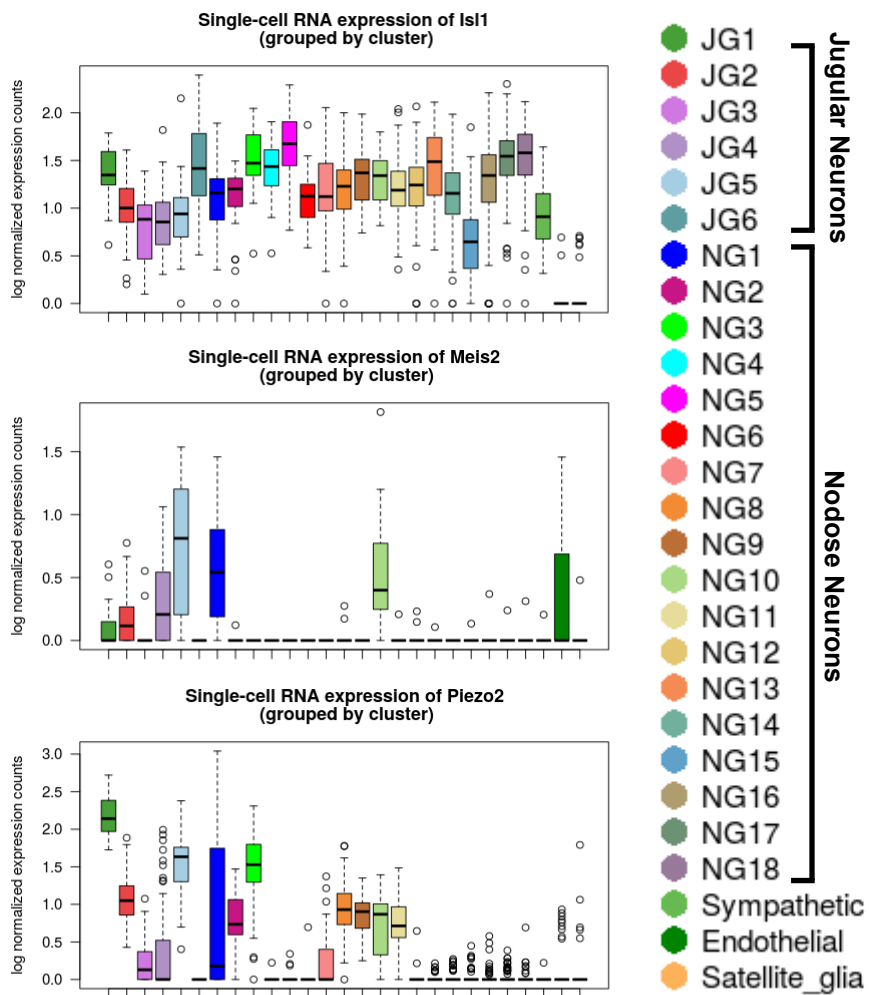

**Figure Supplementary 2**

Supplement: Supplementary file 2 — Supplementary Information 2. [file 41598_2022_24616_MOESM2_ESM.pdf]
