# Supplementary material for: The autism-associated Meis2 gene is necessary for cardiac baroreflex regulation in mice
Source: Sci Rep. 2022 Nov 23;12:20150. doi: 10.1038/s41598-022-24616-5 (PMC9684552; doi:10.1038/s41598-022-24616-5)
Supplement: Supplementary file 3 — Supplementary Legends. [file 41598_2022_24616_MOESM3_ESM.docx]

**Supplementary Figure 1:** Representative traces showing the mean blood arterial pressure (MAP) and related heart rate recording during pharmacological injection of Nitroprusside (A and B), Norepinephrine (C and D), and Phenylephrine (E and F) in anesthetized WT (A, C and E) and Isl1^+/CRE^::Meis2^LoxP/LoxP^ mice (B, D and F).

**Supplementary Figure 2:** Graphs showing Islet1, Meis2 and Piezo2 expression extracted from scRNAseq of jugular and nodose ganglia (Data were extracted from https://ernforsgroup.shinyapps.io/vagalsensoryneurons/). Note that Islet1 is expressed by all neuronal populations ensuring recombination in all vagal neurons. Meis2 is expressed by 5 out of the 6 clusters of jugular neurons and in 2 of the 18 clusters of nodose neurons. In all those clusters, neurons also express Piezo2.
